# The association of FKBP5 gene polymorphism with genetic susceptibility to depression and response to antidepressant treatment- a systematic review

**DOI:** 10.1186/s12888-024-05717-z

**Published:** 2024-04-12

**Authors:** Ying Zhang, Weihua Yue, Jie Li

**Affiliations:** 1 Institute of Mental Health, Peking University Sixth Hospital, 100191 Beijing, China; 2 Tianjin Anding Hospital, Tianjin Municipal Mental Health Center, 300222 Tianjin, China; 3 National Clinical Research Center for Mental Disorders, Peking University Sixth Hospital), 100191 Beijing, China; 4 NHC Key Laboratory of Mental Health, Peking University, 100191 Beijing, China; 5 PKU-IDG/McGovern Institute for Brain Research, Peking University, 100871 Beijing, China; 6 Chinese Institute for Brain Research, 102206 Beijing, China; 7 Institute of Mental Health, Tianjin Anding Hospital, Mental Health Center of Tianjin Medical University, 300222 Tianjin, China

**Keywords:** Depression, FKBP5, Polymorphism, Genetic susceptibility, Antidepressant response

## Abstract

**Background:**

Given the inconsistencies in current studies regarding the impact of FKBP5 gene polymorphisms on depression, arising from variations in study methods, subjects, and treatment strategies, this paper provides a comprehensive review of the relationship between FKBP5 gene polymorphisms and genetic susceptibility to depression, as well as their influence on response to antidepressant treatment.

**Methods:**

Electronic databases were searched up to April 11, 2023, for all literature in English and Chinese on depression, FKBP5 gene polymorphisms, and antidepressant treatment. Data extraction and quality assessment were performed for key study characteristics. Qualitative methods were used to synthesize the study results.

**Results:**

A total of 21 studies were included, with the majority exhibiting average to moderate quality. Six SNPs (rs3800373, rs1360780, rs9470080, rs4713916, rs9296158, rs9394309) were broadly implicated in susceptibility to depression, while rs1360780 and rs3800373 were linked to antidepressant treatment sensitivity. Additionally, rs1360780 was associated with adverse reactions to antidepressant drug treatment. However, these associations were largely unconfirmed in replication studies.

**Conclusions:**

Depression is recognized as a polygenic genetic disorder, with multiple genes contributing, each exerting relatively small effects. Future studies should explore not only multiple gene interactions but also epigenetic changes. Presently, research on FKBP5 in affective disorders remains notably limited, highlighting the necessity for further investigations in this domain.

## Introduction

Depression is a widespread disorder affecting an increasing number of individuals globally, emerging as a significant global health concern. It currently stands as the third leading cause of morbidity in terms of disability-adjusted life years and is projected to become the primary cause by 2030 [1–3]. The clinical manifestation and development of this disorder are influenced by various factors, encompassing genetic, biological, psychosocial, and environmental dimensions [4–7]. Research has found that depression has significant genetic components, and genes play an important role in its onset. Multiple studies have shown that individuals with a family history of depression have a significantly increased risk of developing the condition [8]. Approximately 40% of the risk associated with developing depression is attributed to genetic variants [8]. The twin study estimated that the heritability of depression is about 37%, but when considering factors such as severity, recurrence, and age of onset, this number increases to 70% [9]. Genetic and epigenetic factors play a role in the progression and treatment of depression, as evidenced by numerous studies in this field [10–12]. There are significant differences in individual responses to antidepressant drugs, some of which can be attributed to genetic factors. Research has demonstrated that genetic variations can affect the absorption, distribution, metabolism, and target sensitivity of antidepressants [13]. The initial response rate to antidepressant treatment is approximately 50%, with a subsequent depression remission rate of around 37% [14]. The response of patients to antidepressants is viewed as a polygenic trait, with common genetic variants contributing to more than 40% of the variability in response [15]. Inter-patient variations in response to and efficacy of antidepressant medication can be ascribed to a complex interplay of environmental, physiological, and psychological factors, along with comorbidities and genetic variants. Moreover, contemporary psychotropic medications (encompassing antidepressants, antipsychotics, and mood stabilizers) may operate, at least in part, by inducing epigenetic changes [16].

FKBP5, located on chromosome 6 (OMIM/location: 6p21.3-21.2, full gene length 154,999 bp), is a protein-coding gene characterized by multiple single nucleotide polymorphisms (SNPs) [17]. Functionally, FKBP5 predominantly regulates the glucocorticoid receptor (GR) via two key mechanisms: hormone binding and nuclear translocation. Notably, overexpression of FKBP5 results in reduced GR nuclear transcription and hormone levels [18]. Acting as a co-chaperone alongside heat shock protein 90 (Hsp90), FKBP5 modulates the sensitivity of the GR. Its interaction with the receptor complex leads to reduced cortisol binding and less efficient nuclear translocation of the receptor. Activation of GR induces FKBP5 mRNA and protein expression through intronic hormone response elements, establishing an ultrashort feedback loop that influences GR sensitivity. In the maturation process of the GR complex, FKBP5 binds to Hsp90 through a tetratricopeptide repeat protein (TPR) domain, functioning as a docking station for various co-chaperonins. In this conformation, the receptor complex exhibits reduced resistance to cortisol. Following hormone binding, FKBP5 is exchanged with another TPR-containing immunophilin, FKBP4. The recruitment of dynamin by FKBP4 facilitates the complex’s nuclear translocation and subsequent transcriptional activity [18]. Polymorphisms in the FKBP5 gene are associated with intracellular FKBP5 protein expression, influencing GR alterations and modulating the hypothalamic-pituitary-adrenal (HPA) axis dynamics [19]. Dysfunction of the HPA axis is implicated in the pathogenesis of depression and may significantly impact the response to pharmacotherapy [20]. Research indicates that 50–70% of individuals with depression exhibit dysregulation of the HPA axis [21–23]. Certain individuals with Major Depressive Disorder (MDD) display persistent elevation in HPA activity, with multiple studies reporting abnormalities in cortisol suppression [24]. In chronic MDD (symptoms lasting longer than two years), HPA reactivity does not seem to be significantly affected. Research indicates a direct proportionality between the severity of depressive symptoms and cortisol levels. Additionally, the cortisol response in patients with atypical MDD closely resembles that observed in healthy controls [25]. Hence, FKBP5 has emerged as a focal point in the field of depression genetics research.

Determining the impact of polymorphisms in the FKBP5 gene on genetic susceptibility to depression and their influence on the response to antidepressant medication could enable the prediction of treatment effectiveness and medication side effects for a patient prior to initiating treatment. Yet, the present studies exhibit variations in their findings attributed to differences in methodologies, study populations, clinical phenotypes, and treatment strategies. This review aims to address three key questions: firstly, which FKBP5 gene polymorphisms differentiate depressed patients from healthy populations before treatment initiation; secondly, which FKBP5 gene polymorphisms predict treatment response to antidepressants before treatment initiation; and thirdly, which FKBP5 gene polymorphisms prospectively evaluate adverse effects following antidepressant treatment.

## Materials and methods

We adhered to the Preferred Reporting Items for Systematic Reviews and Meta-Analyses (PRISMA) guidelines for reporting this meta-analytic review. A study protocol with the registration number CRD42024502944 was submitted to PROSPERO (International Prospective Register of Systematic Reviews) before conducting the final analysis for this review.

### Search strategy

For a comprehensive and systematic literature review, both computerized and manual searches were employed to identify relevant studies. Initially, an extensive search was carried out across eight databases, namely PubMed, EMBASE, CBM, CNKI, Wan Fang, VIP, Web of Science, and the Cochrane Library. The literature search involved various combinations of terms such as “affective,” “depression,” “mood,” “FKBP5,” along with “antidepressant,” “tricyclic,” “SSRI,” and “SNRI.” This search encompassed articles available up to April 11, 2023. Furthermore, manual searches were conducted on the reference lists of the chosen articles and relevant review articles on the subject.

### Eligibility criteria

Inclusion criteria: (1) the study involved patients with confirmed depression; (2) it analyzed the relationship between FKBP5 gene polymorphisms and susceptibility to depression or response to antidepressant treatment; (3) the article was published in peer-reviewed English or Chinese journals with full-text availability; (4) it explored genetic variants in human biological specimens. Exclusion criteria encompassed reviews, systematic reviews, commentaries, animal studies, books, or any published material not classified as original research. Extracted information included authors, year of publication, location/ethnicity, study design, sample size, type of depression, type of antidepressant, SNPs, and analysis results.

### Study selection

**Fig. 1:**
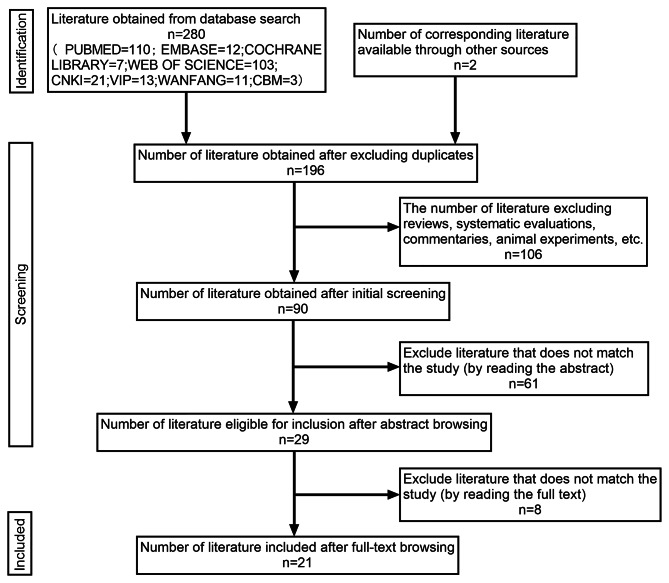
Flow diagram showing the search, article selection, and extraction process

Initially, 280 articles were identified through the screening process. A manual examination of the references of these articles uncovered two additional studies, resulting in a total of 196 articles after eliminating duplicate studies and data. Following the exclusion of reviews, systematic evaluations, commentaries, and animal experiments, the count reduced to 90 articles. Upon reviewing titles and abstracts, 61 articles that did not meet the inclusion criteria were excluded, leaving a total of 29 articles. Following a thorough examination of the full text, an additional eight articles failing to meet the inclusion criteria were excluded. Subsequently, 21 studies were ultimately selected for a comprehensive review and quality assessment. The literature screening process is shown in Fig. 1.

### Characteristics of the included studies

The included studies encompassed diverse ethnicities, settings, and employed varying diagnostic criteria and assessment methods. Table 1 presents the fundamental characteristics of these studies.

**Table 1:** Basic characteristics of the studies

First author	Year	Country	Study design	Research Subjects	Ethnic	Sample Size	Gender (M/F)	Age, years (range, mean)	Sample resource	Diagnostic criteria	
Yang dong	2013	China	Case-control study	MDD	Han	263	Response 89/92	31 ± 11	Outpatient	DSM-IV	
							Non-response 29/18	31 ± 10			
Yu yan	2012	China	Case-control study	Depression	Han	254	126/128	31 ± 11	Outpatient	DSM-IV	
Binder, E. B.	2004	Germany	Case-control study	Unipolar depression	Caucasians	Original sample 294	122/172	47.65 ± 14.5	Inpatient	DSM-IV	
				BD							
				PDD		replication sample 85	29/56	50.5 ± 12.48			
Brent, D.	2010	United States	cohort study	MDD for adolescents	European origin	155	46/109	15.7 ± 1.6	-	DSM-IV	
Cattaneo, A.	2013	Europe	Open, partially randomized, multicenter pharmacogenetics study	Moderate-to-severe unipolar depression	White Europeans	74	31/43	38.3 ± 10.9	Outpatient	ICD-10	
										DSM-IV	
Dam, H.	2019	Denmark	Case-control study	Unipolar depression	85% Danish	718	227/491	-	Danish Psychiatric Biobank	ICD-10	
Ellsworth, K. A.	2013	United States	Case-control study	Depression	Mayo: 337 Non-Hispanic White、96 African Americans、96 Han Chinese Americans	529	-	-	Mayo PGRN-AMPS	DSM-IV	
					STAR*D replication sample: Non-Hispanic White	960			STAR*D		
Fabbri, C.	2018	Europe	Open multicenter study	ES1: MDD	345 Caucasians	357	103/254	51.25 ± 14.51	Inpatient、Outpatient	DSM-IV	
				ES2: Moderate-to-severe depression	208 Caucasians	218	72/146	47 ± 12.56	-		
				ITAS: Non-psychotic MDD	-	96	32/64	57.34 ± 15.97	Outpatient		
				STAR*D replication sample response: -	-	1409	565/844	42.94 ± 13.49	STAR*D		
				STAR*D replication sample remission: -	-	620	264/356	43.11 ± 12.95			
Gawlik M	2006	Germany	Case-control study	Recurrent depression	Caucasians	57	32/25	48.1	-	ICD-10	
				BD		191	122/69				
Ising, M.	2019	Germany	Naturalistic Open Label Longitudinal Treatment Study	Moderate-to-severe depressive disorder	-	297	Response 96/77	48.8 ± 14.0	Inpatient	ICD-10	
							Non-response 61/63	47.0 ± 13.4			
Kirchheiner, J.	2008	Germany	cohort study	Unipolar depression	Caucasians	179	61/118	45.92 ± 11.73	Inpatient	ICD-10	
				BD						DSM-IV	
Lekman, M.	2008	United States	Case-control study	Non-psychotic MDD	1256 Non-Hispanic White	1523(1809 for genetic analysis)	-	-	STAR*D outpatient sample	DSM-IV	
					267 Black						
Nobile, B.	2020	France	Prospective cohort study	MDD	Caucasians	496	192/304	48.05 ± 14.75	Outpatient	DSM-IV	
Papiol, S.	2007	Spain	Prospective cohort study	Depression	Spanish	159	35/124	39.5 ± 12.2	Outpatient	DSM-IV	
Perroud, N.	2011	Switzerland	Partially randomized study	Moderate-to-severe depressive disorder	95 European origin	131	53/78	36.65 ± 10.69	Outpatient	ICD-10	
										DSM-IV	
Sarginson, J. E.	2010	United States	RCT	MDD in the elderly	226 Caucasians	246	Paroxetine 57/65	72.25 ± 5.13	Outpatient	DSM-IV	
							mirtazapine 63/61	71.91 ± 5.67			
Stamm, T. J.	2016	Germany	RCT	MDD	Central European origin	298	111/187	44.35 ± 12.04	Inpatient	DSM-IV	
Szczepankiewicz, A.	2014	Poland	Case-control study	BD	Polish	528	221/307	44.36 ± 13.9	Inpatient	DSM-IV	
				MDD		218	50/168	45.5 ± 14.0			
Tsai, S. J.	2007	China	Prospective cohort study	MDD	Ethnic Chinese	125	56/69	42.1 ± 16.2	Outpatient	DSM-IV	
				PDD							
Yang, C.	2021	China	Case-control study	Depression	Han	181	TRDI:47/34	46.0 ± 12.7	Inpatient、Outpatient	DSM-IV	
							MDNTR:24/76	42.8 ± 10.2			
Zobel, A.	2010	Germany	Case-control study	Recurrent unipolar depression	Caucasians	268	98/170	48.92 ± 14.0	Inpatient	DSM-IV	

First author	Assessment Scale	AD	SNPs	Genotype method	Definition of treatment response	Assessment time	Results	
Yang dong	HAMD-17	escitalopram	rs3800373	PCR-LDR	Response: HAMD score reduction rate < 50%	Week 6	rs3800373、rs1360780 unable to predict treatment response to antidepressants	
		Paroxetine	rs1360780					
	TESS	Venlafaxine	rs1360780		No response: HAMD score reduction rate ≥ 50%			
		Duloxetine						
Yu yan	HAMD-17	-	rs3800373	PCR-LDR	-	-	Lack of association between rs3800373、rs1360780 and susceptibility to depression	
	HAMA		rs1360780					
	BPRS							
Binder, E. B.	HAMD-21	SSRIs	rs3800373	MALDI-TOF-MS Real-time PCR	Response: a decrease > 50% in the HAMD score at week 5	Week 2、5	rs1360780 TT genotype responded faster and better to antidepressants, rs3800373 CC genotype responded better to treatment than other genotype carriers, and there was no difference in the frequency of individual SNPs or haplotypes between depressed patients and healthy controls	
		TCAs	rs1360780					
	MADRS	mirtazapine	rs4713916		Remission: HAMD ≤ 10			
		Reboxetine						
Brent, D.	CDRS	Fluoxetine	rs1360780 rs3800373	fluorescence polarization method	Response: a decrease > 50% in the CDRS score and a score < 40 at week 12	Week 6、12	rs1360780 TT and rs3800373 GG genotypes were associated with higher and earlier occurrence of suicidal events, and there was no significant relationship between genotype and treatment response	
	CGI	Paroxetine						
	C-SSRS	citalopram						
		Venlafaxine						
Cattaneo, A.	MADRS HAMD-17	escitalopram	-	Real-time PCR	Response: a reduction in MADRS > 50% at week 12	Week 8、12	Successful antidepressant response is associated with reduced levels of FKBP5, a gene that does not predict response to antidepressants	
	BDI	Nortriptyline						
Dam, H.	-	-	rs1360780	Real-time PCR	-	-	rs1360780 is not associated with susceptibility to depression and the CC genotype has a higher family history of depression	
				TaqMan				
Ellsworth, K. A.	QIDS-16	citalopram	384个SNPs	Sanger	Response: ≥ 50% reduction in QIDS score	Mayo: Week 8 STAR*D: Week 6	rs1360780、rs3800373 and rs4713916 were not associated with SSRI treatment outcomes in MDD, and rs352428 was associated with 8-week treatment response in the Mayo study and 6-week treatment response in the STAR*D replication study	
	HAMD	escitalopram		Illumina/Affymetrix	Remission: a QIDS score of ≤ 5 at the last visit			
				TaqMan				
Fabbri, C.	HAMD-21	Venlafaxine	rs9296157 rs9470080 rs1043805 rs3800373 rs1360780 rs4713916 rs3800373 rs352428	Real-time PCR	Response: a decrease of at least 50% in the HDRS-21 or the MADRS or the QIDS-C at week 4 or 6	Week 4	rs3800373 in ES1 the CC genotype had a high risk of treatment non-response, in ES2 and ITAS the AA genotype and A allele had better response and remission. rs1360780 in ES2 the CC genotype and C allele were associated with better response and remission to venlafaxine and a reduced risk of TRDA, in ITAS the CC genotype and C allele were associated with better response and remission. In STAR*D replication samples rs9368882 was associated with level 1 remission	
	MADRS	citalopram				Week 12		
	HAMD-21	bupropion-SR				Week 8		
	QIDS	Sertraline			Remission: HDRS ≤ 7 or MADRS < 10 or QIDS-C ≤ 5	Week 2、4、6、9、12		
		escitalopram						
Gawlik M	-	-	rs4713916 rs1360780 rs3800373	PCR	-	-	FKBP5 polymorphisms and haplotypes are not associated with the inheritance of affective disorders	
				TaqMan				
Ising, M.	HAMD-21	SSRIs	rs1360780	Illumina Beadchip technology	Response: a reduction of the HAMD-21 score of at least 50%	Week 6	Successful antidepressant treatment outcomes were accompanied by reduced expression of FKBP5 gene and FKBP51 protein, with the rs1360780 T allele showing a better treatment response	
		TCAs						
		SNRIs		Real-time PCR				
		NaSSA		TaqMan				
		Other						
Kirchheiner, J.	HAMD-21	SSRIs	rs3800373 rs1360780	Real-time PCR	Response: HDRS decreased by ≥ 50% at day 21	Week 3	A higher proportion of rs3800373 AA genotype carriers were diagnosed with bipolar disorder, and patients carrying rs1360780 CC genotype or rs3800373 AA genotype were poorly treated with antidepressants, with a closer association with venlafaxine treatment efficacy	
		mirtazapine						
		Venlafaxine		TaqMan				
		TcAs						
		Other						
Lekman, M.	QIDS	citalopram	rs1360780 rs4713916 rs3800373	Illumina	Remission: QIDS-C16 ≤ 5	Week 2、4、6、9、12、14	rs1360780 was significantly associated with disease status in non-Hispanic whites but not in blacks. rs1360780 and rs4713916 were strongly LD in non-Hispanic whites but not in blacks.When all races were analyzed together rs4713916 was significantly associated with remission, but not with response, and this association was primarily caused by non-Hispanic whites. No correlation was detected between rs3800373、 rs1360780 and antidepressant response	
				TaqMan	Response: QIDS-C16 reduced by ≥ 50%			
Nobile, B.	HADS	Tianeptine	rs3800373 rs7757037 rs737054 rs1360780 rs9470080 rs6902321	Real-time PCR	TESI: having a MADRS-SI score of 0 or 1 at baseline, followed by a score > 1 at least once during the follow-up	Week 2、4、6	rs6902321 TT genotype is significantly associated with treatment-triggered suicidal ideation, and no SNP is associated with TWOSI	
	MADRS-SI			TaqMan	TWOSI: the worsening of pre-existing SI when starting a new antidepressant drug			
Papiol, S.	HAMD-21	citalopram	rs1360780	Applied Biosystems SNaP-Shot	Response: HDRS at 4th week ≤ 50%	Week 4、8、12	rs1360780 TT genotype has poor antidepressant response to citalopram, and FKBP5 genotype is not a predictor of treatment outcome	
				TaqMan	Remission: HDRS at 12th week ≤ 7			
Perroud, N.	MADRS	Paroxetine	rs1360780	-	Increasing suicidal ideation: Any 1-point increase which reached a level of 2 at least on the 10th suicidal item of the MADRS	Assessed every 2 weeks for 30 weeks	Carriers of the rs1360780 T allele have a higher risk of increased suicidal ideation on treatment, and there is no association between rs1360780 and drug blood levels	
		Venlafaxine						
		clomipramine						
		nefazodone						
Sarginson, J. E.	HAMD-17	Paroxetine	rs1360780 rs3800373	TaqMan	Remission: HDRS-17 ≤ 7 or HDRS-21 ≤ 10	Week 1–4、6、8	No association between rs1360780, rs3800373 and clinical outcomes and no prediction of remission or time to remission	
	HAMD-21	mirtazapine			Response: 50% reduction in HDRS-21			
Stamm, T. J.	HAMD-21	Venlafaxine	rs1360780	Real-time PCR	Remission: HDRS-21 < 10	Week 2、4、6、8、10、12、14	The rs1360780 TT genotype showed superior treatment response under all treatment conditions, and the effect of genotype on treatment outcome did not differ significantly between antidepressants. Standardized, quality-controlled treatment can compensate to some extent for the "genetic disadvantage" of C-allele carriers	
		Sertraline						
		Amitriptyline		TaqMan				
		Reboxetine						
Szczepankiewicz, A.	-	-	rs1360780 rs755658 rs9470080 rs4713916 rs7748266 rs9296158 rs9394309 rs3800373	TaqMan	-	-	Associations between rs1360780, rs9470080, rs4713916, rs9296158 and rs9394309 and MDD but not bipolar disorder	
Tsai, S. J.	HAMD-21	Fluoxetine	rs1360780	PCR	Response: ≥50% reduction in HAMD at week 4	Week 4	No association between rs1360780 genotype and short-term antidepressant treatment response and lifetime depressive episodes	
Yang, C.	HAMD-17	-	rs1043805 rs3800373 rs9296158 rs7748266 rs1360780 rs2766537 rs9394309 rs9470080 rs2817035	MALDI-TOF MS	TRDI: CRP 0. 85 − 10 mg/L;Insufficient response to ≥ 1 antidepressants, at least 6w and adequate dose; Current antidepressant treatment ≥ 2w	-	No association of SNPs and haplotype combinations of FKBP5 with MDD or antidepressant treatment response	
Zobel, A.	HAMD	citalopram	rs3800373 rs755658 rs1360780 rs1334894 rs4713916	TaqMan	-	Week 4	rs3800373 AA, rs4713916 GG (related to clinical diagnosis) showed less reduction in cortisol secretion in the Dex/CRH test after 4 weeks of citalopram treatment	

Note: ES: European sample; ITAS Italian sample STAR*D Sequenced Treatment Alternatives to Relieve Depression Mayo PGRN-AMPS: Mayo Clinic Pharmacogenomics Research Network-Antidepressant Medication Pharmacogenomic Study; MDD: Major Depressive Disorder; BD: Bipolar disorder; PDD: Persistent depressive disorder; TRDI: antidepressant treatment resistance and increased inflammatory activity; MDNTR: major depression without treatment resistance; DSM-IV: Diagnostic and Statistical Manual of Mental Disorders, Fourth Edition; ICD-10: international Classification of diseases-10

Note: AD: antidepressive drugs；SNPs：single nucleotide polymorphisms；HAMD：Hamilton depression scale；HDRS：Hamilton Depression Rating Score；HADS：Hospital Anxiety and Depression Scale；MADRS：Montgomery-Asberg Depression Rating Scale；QIDS：Quick Inventory of Depressive Symptomatology；BDI：Beck Depression Inventory；CDRS：Children’s Depression Rating Scale；CGI：Clinical Global Impressions scale；C-SSRS：Columbia-Suicide Severity Rating Scale；BPRS：Brief Psychiatric Rating Scale；HAMA：Hamilton Anxiety Scale；TESS：Treatment Emergent Symptom Scale；SSRIs：selective serotonin reuptake inhibitor；TCAs：Tricyclic Antidepressive Agents；SNRIs：serotonin-norepinephrine reuptake inhibitor；NaSSA：Noradrenergic and specific serotonergic antidepressant；TESI：treatment-emergent suicidal ideation；TWOSI：treatment worsening of suicidal ideation；TRDI: antidepressant treatment resistance and increased inflammatory activity;

### Assessment of study quality

The included studies exhibited considerable heterogeneity across various characteristics, including study subjects, methods, SNPs, diagnostic criteria, assessment scales, type and dose of antidepressants, and the definition of treatment response. Due to this high heterogeneity, the use of Meta-analysis for all included studies in this review was precluded. Consequently, this paper adopts a qualitative approach to summarize the relationship between FKBP5 gene polymorphisms and genetic susceptibility to depression and antidepressant treatment response.

Tables 2 and 3 display the quality assessment of the 21 included studies. 19 case-control and cohort studies, along with inconveniently classified studies, were assessed for quality using the NOS scale. Two RCT studies underwent quality evaluation using the modified Jadad scale. None of the studies fulfilled all quality criteria, with the majority being classified as moderate quality.

**Table 2:** Quality assessment of NOS

Study		Selection	Comparability	Outcome	Total Score
Yang dong	2013	**	*	*	****
Yu yan	2012	***	*	*	*****
Binder, E. B.	2004	****	*	*	******
Brent, D.	2010	**	*	*	****
Cattaneo, A.	2013	****	*	*	******
Dam, H.	2019	***	-	*	****
Ellsworth, K. A.	2013	**	-	*	***
Fabbri, C.	2018	**	-	*	***
Gawlik M	2006	****	*	*	******
Ising, M.	2019	***	*	*	*****
Kirchheiner, J.	2008	**	-	*	***
Lekman, M.	2008	****	*	*	******
Nobile, B.	2020	***	*	**	******
Papiol, S.	2007	****	-	*	*****
Perroud, N.	2011	**	-	**	****
Szczepankiewicz, A.	2014	****	-	*	*****
Tsai, S. J.	2007	**	-	*	***
Yang, C.	2021	***	-	**	*****
Zobel, A.	2010	****	**	**	********

Notes: In this meta-analysis, only studies with a total score of 3 or higher will be included

**Table 3:** Quality assessment of Modified Jadad Scale

Study		Randomization	Concealment of allocation	Double blinding	Withdrawals and dropouts	Total Score
Sarginson, J. E.	2010	1	1	1	1	4
Stamm, T. J.	2016	1	1	2	1	5

Notes: In this meta-analysis, only studies with a total score of 3 or higher will be included

### Statistical analysis

Data Analysis The data were analyzed using Review Manager 5.4.1 software. Individual and pooled odds ratios (OR) with their associated 95% confidence intervals (CIs) were calculated. Significance of the pooled effect size was determined through a Z test. Heterogeneity across studies was assessed using the *χ*^*2*^-test of fit and I^*2*^ measure. Outcome Measure Response, defined as a ≥ 50% decrease in HAMD or QIDS, was utilized as the primary outcome measure. Data Extraction and Subgroup Analysis Six studies addressing treatment response provided raw data. Initially, data were pooled for analysis across all races, followed by separate analyses for Caucasians and Asians, considering the distinct distribution of genotype frequencies across racial groups. Sensitivity analyses were conducted to mitigate the potential impact of individual studies on the final results. Assessment of Publication Bias Publication bias was evaluated through the generation of funnel plots.

## Results

The original studies within these 21 literature sources investigated 384 SNPs in the FKBP5 gene. 17 of these SNPs explicitly presented study outcomes (rs1043805, rs1334894, rs1360780, rs2766537, rs2817035, rs3800373, rs4713916, rs6902321, rs737054, rs755658, rs7748266, rs7757037, rs9296157, rs9296158, rs9368882, rs9394309, rs9470080), with a predominant focus on three SNPs (rs3800373, rs1360780, rs4713916). The publication dates of these articles span from 2004 to 2021, predominantly conducted in European countries, involving individuals of Caucasian or European descent, and employing case-control or cohort study designs. Among the included studies, 12 articles investigated the association between FKBP5 gene polymorphisms and genetic susceptibility to depression. Additionally, 17 articles explored the association between FKBP5 gene polymorphisms and sensitivity to antidepressant treatment, while four articles focused on the association between FKBP5 gene polymorphisms and adverse effects induced by antidepressant treatment.

### FKBP5 gene polymorphism and genetic susceptibility to depression

The findings revealed associations between six SNPs (rs3800373, rs1360780, rs9470080, rs4713916, rs9296158, rs9394309) and genetic susceptibility to depression [26–28]. Additionally, depressed patients exhibited higher FKBP5 mRNA expression levels compared to healthy controls [29]. Nevertheless, the alleles and genotypes linked to genetic susceptibility to depression exhibited inconsistencies across studies. FKBP5 gene polymorphisms demonstrated an association with susceptibility to depression in studies conducted in Poland, Italy, Germany, the United States, and certain European countries [26, 27, 30, 31]. In contrast, FKBP5 gene polymorphisms did not exhibit an association with susceptibility to depression in some studies from Germany, Denmark, China, and studies involving black subjects [17, 30, 32–37].

### FKBP5 gene polymorphism and antidepressant treatment sensitivity

**Fig. 2:**
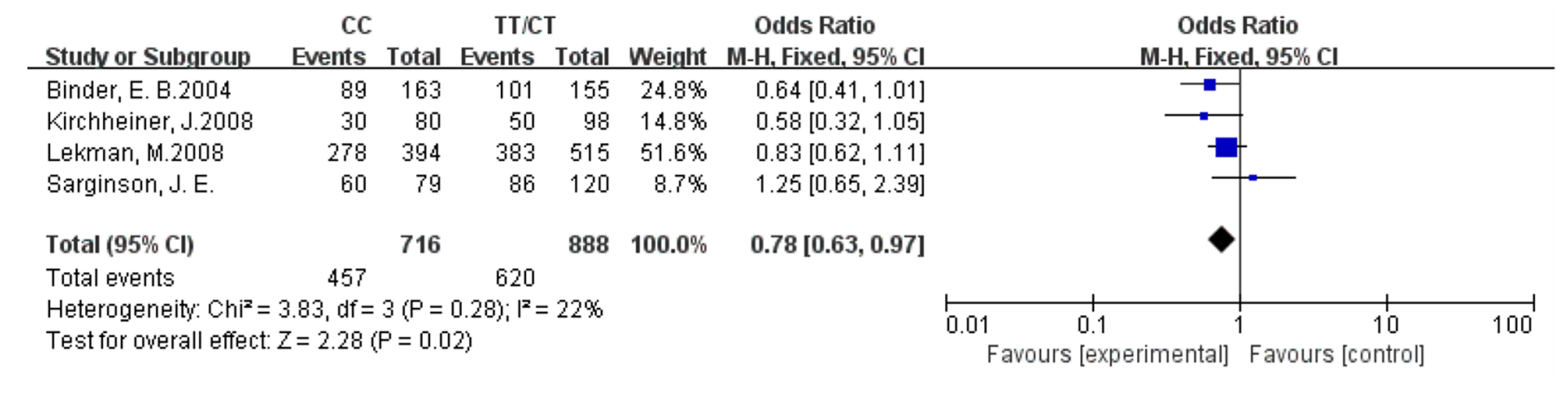
The relationship between rs1360780 and treatment sensitivity in Caucasians (white) was examined, comparing the CC genotype with the TT and CT genotypes. A Forest Plot of studies illustrates this relationship

**Fig. 3:**
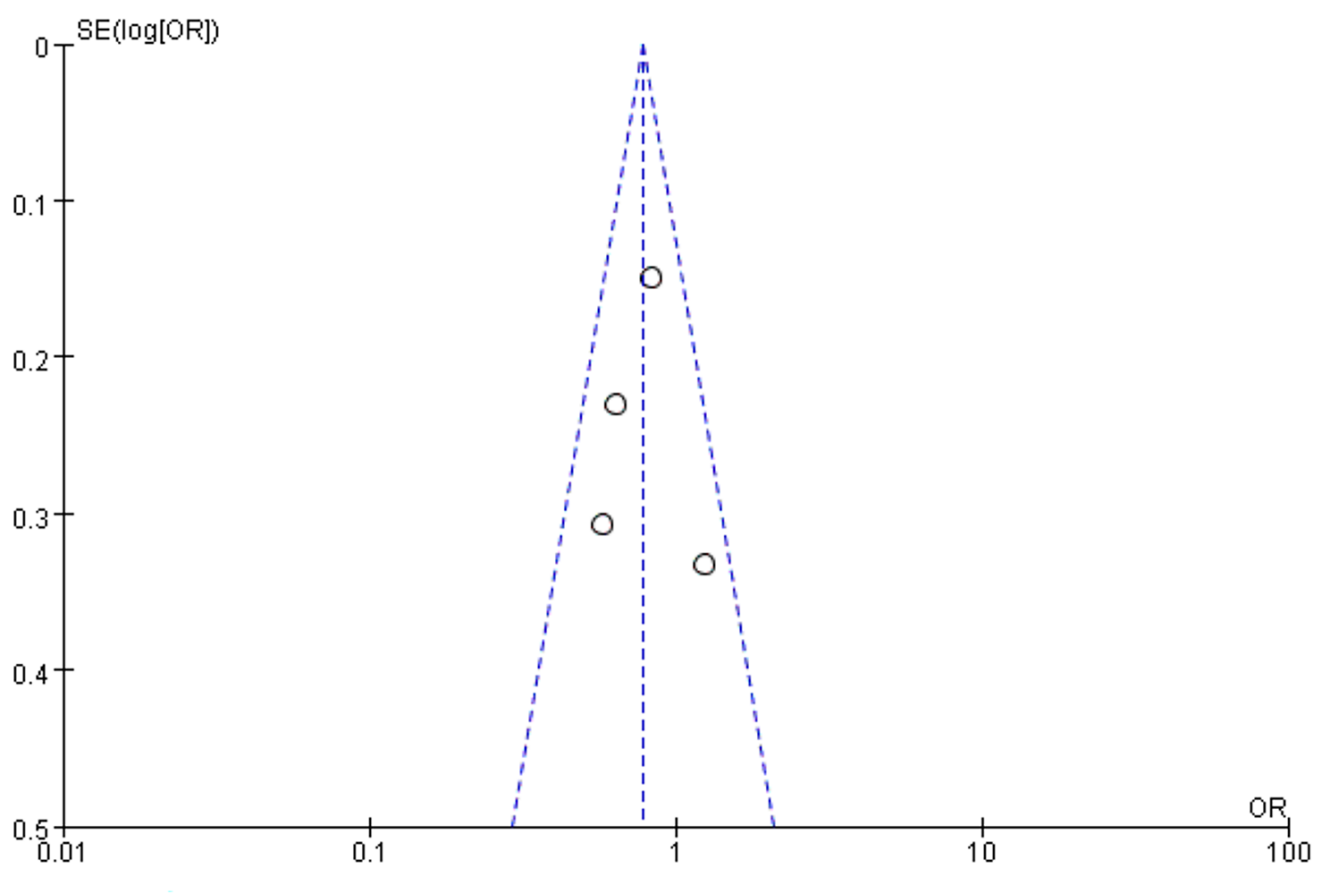
The relationship between rs1360780 and treatment sensitivity in Caucasians (white) was investigated by comparing the CC genotype with the TT and CT genotypes. A Funnel Plot of the comparison is presented

In the present study, the most prominent SNPs (rs1360780, rs3800373, rs4713916) and antidepressants (citalopram, escitalopram, paroxetine, venlafaxine) were examined. Depression is characterized by concurrent higher FKBP5 mRNA expression and lower GR levels, resulting in GR resistance. Successful antidepressant treatment necessitates normal GR function [29, 38, 39]. Carriers of the rs1360780 T allele demonstrated enhanced treatment response under all treatment conditions in two German studies [38, 40]. Analysis of the rs1360780 genotype and treatment response across six studies revealed no significant association in the mixed ethnicity analysis [30–32, 36, 41, 42]. However, when analyzed for Caucasians alone, patients carrying the rs1360780 T allele exhibited faster response and better efficacy to antidepressants (OR = 0.78, 95%CI: 0.63–0.97, *P* = 0.02) with low to moderate heterogeneity (I^2^ = 22%) (see Figs. 2 and 3). In the German Caucasian population, carriers of the rs3800373 C allele demonstrated a better treatment response than carriers of other genotypes in two studies [31, 32]. However, the analysis of the association between the rs3800373 genotype in four studies [30–32, 42] and the rs4713916 genotype in two studies [30, 32] did not reveal evidence of mixed ethnicity or Caucasian association. The FKBP5 gene polymorphism did not predict treatment response to antidepressants in studies involving white European, European American, non-Hispanic white, and Chinese Han populations. Furthermore, it was not associated with the type of antidepressant used [29, 34, 36, 37, 40, 41, 43, 44]. The impact of other SNPs of the FKBP5 gene on the response to antidepressant treatment exhibited inconsistencies across studies from diverse environments and ethnicities [27, 28, 30, 35, 42, 44, 45]. None of the studies identified specific genotypes associated with successful treatment using particular antidepressants. Remission was not analyzed in this review due to the limited availability of data from only one study.

### FKBP5 gene polymorphisms and adverse effects caused by antidepressant treatment

Recent investigations into treatment-induced adverse effects have primarily concentrated on the emergence or exacerbation of suicidal ideation during antidepressant treatment. The more extensively studied loci include rs1360780 and rs3800373. Studies have demonstrated that carriers of the rs1360780 T allele face a heightened risk of experiencing increased suicidal ideation during treatment, particularly in studies involving individuals of European ancestry in the United States and Switzerland. This risk is independent of the type of antidepressant used [44, 45]. However, the relationship between other SNPs of FKBP5 and treatment-induced adverse reactions has not been consistently established across studies [17, 46].

### Haplotypes

In studies involving Caucasian and non-Hispanic white populations, rs3800373, rs1360780, and rs4713916 were identified within a haplotype block. This haplotype was associated with an increased risk of depression [28, 30, 33]. Conversely, in black and Chinese Han populations, rs1360780 and rs3800373 are part of a single block, while rs4713916 belongs to a distinct block and is not associated with susceptibility to depression or response to antidepressant treatment [30, 34, 37, 41]. However, there are no consistent findings regarding the haplotype distribution of FKBP5 gene SNPs and their association with depression susceptibility and antidepressant treatment response in other studies [26, 43].

### Linkage disequilibrium (LD)

Numerous SNPs within the FKBP5 locus exhibit high LD in various populations, encompassing Caucasian, non-Hispanic white, black, and Asian populations. Specifically, rs1360780 and rs3800373 demonstrate high LD in non-Hispanic white, black, and Chinese Han populations [30, 34, 37, 44]. In non-Hispanic white populations, rs1360780 and rs4713916 exhibit high LD, whereas this association is not observed in the black population with high LD [30]. However, there are no consistent findings regarding LD status and the relationship with depression among other SNPs across studies.

## Discussion

The primary challenge in genetic association studies lies in the replication of findings [47]. Depression is a polygenic genetic disorder with a complex genetic mechanism. A specific genetic polymorphism may exert its influence through the association of one or several other genetic polymorphisms within the same gene LD. As an illustration, the rs1360780 T allele and the rs3800373 C allele exhibit similar correlations with antidepressant response in Caucasian populations due to their high LD. Furthermore, given the complexity of how depression is inherited and the involvement of numerous genetic loci, it is plausible that genes act in concert to manifest specific clinical symptoms. Polymorphisms at a particular locus may only represent a risk factor for one or several clinical symptoms of depression [48]. Consequently, studies should delve into the interaction of multiple genes.

Inconsistent treatment responses may arise from the presence of LD in the gene under investigation and a functional mutation influencing the efficacy of antidepressants. The extent of LD varies across ethnic groups, leading to inconsistent results in studies conducted across different populations. Alleles are distributed at different frequencies in distinct ethnic groups. For instance, the rs3800373 (G/T) and rs1360780 (C/T) alleles exhibit varied frequencies in Chinese Han, Caucasian, and Black populations. Additionally, rs4713916 has a lower mutation rate in Asian populations, making it less frequently studied in Asian populations. Furthermore, diverse enrollment criteria, efficacy assessment methods, and selected drugs can impact study results. Additionally, environmental factors (and associated epigenetic modifications), drug-drug interactions, hepatic and renal impairment, and compliance issues all play crucial roles in effective prescribing [49]. Pharmacogenetic studies have explored polymorphisms in several candidate genes (including SLC6A4, HTR2A, CYP2C19, CYP2D6, ABCB1, and FKBP5) for guiding antidepressant treatment versus standard treatment, yielding intriguing results [50]. Virtually all antidepressant pharmacogenetic variants exhibit potential pleiotropic effects associated with major depressive disorder, intermediate phenotypes of emotional processes, and brain areas affected by antidepressant treatment [51]. Pharmacogenetics may also contribute to inconsistent treatment responses.

Elevated suicidal ideation could result from treatment-related side effects or a lack of improvement in depressive symptoms due to the poor efficacy of antidepressants. Moreover, in the absence of placebo-controlled data, it cannot be excluded that the observed association of FKBP5 gene polymorphisms with antidepressant response is potentially a pharmacogenetic effect or is linked to patients being in different phases of depressive episodes. Several SNPs within the FKBP5 locus pose challenges in identifying polymorphisms with a singular pathogenic function due to high LD. Additionally, studies exclusively genetically analyzed subjects from their specific regional lineage, substantially diminishing the reproducibility of the findings. Furthermore, certain epigenetic mechanisms can induce alterations at the level of crucial molecules, consequently diminishing the reproducibility of the findings. As an illustration, chronic stress is linked to the hypomethylation of FKBP5. Reduced methylation of FKBP5 results in the upregulation of FK506-binding protein 51, inhibiting the binding of glucocorticoids to GR. This ultimately leads to the inhibition of negative feedback regulation of the HPA axis [52].

While these 21 studies all investigate FKBP5 gene polymorphisms in relation to genetic susceptibility and treatment response to depression, substantial heterogeneity exists in terms of study subjects, design, depression typing, assessment criteria, and antidepressant type. This heterogeneity not only poses challenges for quantitative analysis between the findings but also contributes to inconsistent results. Firstly, the study subjects exhibited diverse gender and age distributions as well as varying ethnicities. Secondly, there were disparities in clinical variables, including depression typing, severity, antidepressant strategy, duration of treatment, and response and remission criteria across studies. The majority of studies permitted patients to receive antidepressant treatment in a natural setting without intervention in treatment strategies, and in some studies, the inclusion of mood stabilizers, antipsychotics, and psychotherapy further complicates the interpretation of results. These studies share several common limitations. Firstly, a common limitation is the small sample size in most studies. Secondly, there is heterogeneity in both the study samples and treatment protocols. Thirdly, many studies lacked control groups, and even those that included controls exhibited substantial heterogeneity in the age and gender distribution of their control groups, leading to a lack of comparability. Especially in studies on suicidal events, the absence of control groups renders it impossible to ascertain whether suicidal events are linked to diagnosis and treatment, whether the emergence or exacerbation of suicidal ideation is attributable to medication, or whether the disease remains unremitted due to inadequate medication doses. Fourthly, these studies predominantly employed peripheral blood samples to analyze FKBP5 gene expression, potentially inadequately reflecting FKBP5 expression in the pituitary or brain.

Limitations of this review include the following: Firstly, genotype data on treatment response were available for only six of these 21 studies, and the absence of genotype data on included polymorphisms may introduce bias. Secondly, only dichotomous outcomes were assessed, and not symptom improvement. Thirdly, possible sources of heterogeneity for inclusion in the studies were not sufficiently examined, and potential confounders were not adjusted for.

## Conclusions

Genetic and molecular studies have advanced our understanding of the biological basis of depression, and findings from neurobiological studies have contributed to enhancing the clinical outcomes of depressed patients [53]. Numerous associations between genes and various phenotypes of depression have been identified [54]. Susceptibility to depression is influenced by the function of multiple genes and their interactions with each other and various environmental factors, with potential impacts from certain epigenetic changes [52]. The demethylation of FKBP5 polymorphisms (rs1360780, rs3800373, rs9470080, and rs4713916) following childhood trauma leads to heightened HPA axis sensitization and a susceptibility to developing MDD [55]. In this review, the 21 studies broadly identified six SNPs (rs3800373, rs1360780, rs9470080, rs4713916, rs9296158, and rs9394309) that may be linked to susceptibility to depression. Additionally, rs1360780 and rs3800373 were associated with antidepressant treatment sensitivity, and rs1360780 was linked to adverse effects induced by antidepressant treatment. However, in most cases, these associations could not be replicated in subsequent studies. While genetics plays a role in the etiology of depression, findings from identical twin studies reveal substantial variability, suggesting the involvement of non-genetic factors [56]. The FKBP5 gene serves as a crucial modulator of HPA axis reactivity and holds a key position in influencing the risk of stress-related disorders. This suggests the potential for studying the FKBP5 gene in relation to depression, especially in subpopulations such as those who have experienced childhood trauma. With the appropriate study population, significant associations are likely to be discovered. Unfortunately, research on the neuroendocrine system-related candidate gene FKBP5 in mood disorders is currently highly limited, highlighting the need for further studies in this area.

## Data Availability

All data generated or analyzed during this study are included in this published article.

## List of abbreviations

SNPs: single nucleotide polymorphisms
GR: glucocorticoid receptor
Hsp90: heat shock protein 90
TPR: tetratricopeptide repeat protein
HPA: hypothalamic-pituitary-adrenal
MDD: Major Depressive Disorder
HAMD: Hamilton depression scale
QIDS: Quick Inventory of Depressive Symptomatology
LD: Linkage disequilibrium

## References

[1] Clark SLHM, Chan RF (2020). A methylation study of long-term depression risk. Mol Psychiatry.

[2] VG F (2020). Pharmacological sex hormone manipulation as a risk model for depression. J Neurosci Res.

[3] Zhdanava M, Pilon D, Ghelerter I, Chow W, Joshi K, Lefebvre P, Sheehan JJ (2021). The prevalence and national burden of treatment-resistant depression and major depressive disorder in the United States. J Clin Psychiatry.

[4] Gharraee BTK, Sheybani F (2019). Prevalence of major depressive disorder in the general population of Iran: a systematic review and meta-analysis. Med J Islam Repub Iran.

[5] Kraus CKB, Lanzenberger R, Zarate CA, Kasper S (2019). Prognosis and improved outcomes in major depression: a review. TranslPsychiatry.

[6] Bialek KCP, Strycharz J, Sliwinski T (2019). Major depressive disorders accompanying autoimmune diseases-response to treatment.Prog. Neuropsychopharmacol Biol Psychiatry.

[7] Lozupone MPF (2020). Social determinants of late-life depression epigenetics. Epigenomics.

[8] Sullivan PF, Neale MC, Kendler KS (2000). Genetic epidemiology of major depression: review and meta-analysis. Am J Psychiatry.

[9] Menke A, Klengel T, Binder EB (2012). Epigenetics, depression and antidepressant treatment. Curr Pharm Des.

[10] Czarny PBK, Zio´lkowska S, Strycharz J, Barszczewska G, Sliwinski T (2021). The importance of epigenetics in diagnostics and treatment of major depressive disorder. J Pers Med.

[11] Zhou JLM, Wang X (2021). Drug response-related DNA methylation changes in schizophrenia, bipolar disorder, and major depressive disorder. Front Neurosci.

[12] Klinger-K¨onig JHJ, Van der Auwera S (2019). Methylation of the FKBP5 gene in association with FKBP5 genotypes, childhood maltreatment and depression. Neuropsychopharmacology.

[13] Uher R, Perroud N, Ng MY, Hauser J, Henigsberg N, Maier W, Mors O, Placentino A, Rietschel M, Souery D (2010). Genome-wide pharmacogenetics of antidepressant response in the GENDEP project. Am J Psychiatry.

[14] Rush AJ, Trivedi MH, Wisniewski SR, Nierenberg AA, Stewart JW, Warden D, Niederehe G, Thase ME, Lavori PW, Lebowitz BD (2006). Acute and longer-term outcomes in depressed outpatients requiring one or several treatment steps: a STAR*D report. Am J Psychiatry.

[15] Tansey KE, Guipponi M, Hu X, Domenici E, Lewis G, Malafosse A, Wendland JR, Lewis CM, McGuffin P, Uher R (2013). Contribution of common genetic variants to antidepressant response. Biol Psychiatry.

[16] van Westrhenen R, Aitchison KJ, Ingelman-Sundberg M, Jukić MM (2020). Pharmacogenomics of antidepressant and antipsychotic treatment: how far have we got and where are we going?. Front Psychiatry.

[17] Dam H, Buch DJO, Nielsen BA, Weikop P, Werge, Thomas J (2019). Clinical association to FKBP5 rs1360780 in patients with depression. Psychiatr Genet.

[18] Binder EB (2009). The role of FKBP5, a co-chaperone of the glucocorticoid receptor in the pathogenesis and therapy of affective and anxiety disorders. Psychoneuroendocrinology.

[19] Alshaya DS (2022). Genetic and epigenetic factors associated with depression: an updated overview. Saudi J Biol Sci.

[20] Farrell CDK, Oleary N (2018). DNA methylation differences at the glucocorticoid receptor gene in depression are related to functional alterations in hypothalamic-pituitary-adrenal axis activity and to early life emotional abuse. Psychiatry Res.

[21] Watanuki TFH, Uchida S, Matsubara T, Kobayashi A, Wakabayashi Y (2008). Increased expression of splicing factor SRp20 mRNA in bipolar disorder patients. J Affect Disord.

[22] Cherian KSAF, Keller J (2019). HPA axis in psychotic major depression and schizophrenia spectrum disorders: Cortisol, clinical symptomatology, and cognition. Schizophr Res.

[23] Alex FJC, Javier L (2018). FKBP5 polymorphisms and hypothalamic-pituitary-adrenal axis negative feedback in major depression and obsessive-compulsive disorder. J Psychiatr Res.

[24] Nandam LS, Brazel M, Zhou M, Jhaveri DJ (2019). Cortisol and major depressive disorder-translating findings from humans to animal models and back. Front Psychiatry.

[25] Zobel AW, Nickel T, Sonntag A, Uhr M, Holsboer F, Ising M (2001). Cortisol response in the combined dexamethasone/CRH test as predictor of relapse in patients with remitted depression. A prospective study. J Psychiatr Res.

[26] Szczepankiewicz A, Leszczynska-Rodziewicz A, Pawlak J, Narozna B, Rajewska-Rager A, Wilkosc M, Zaremba D, Maciukiewicz M, Twarowska-Hauser J (2014). FKBP5 polymorphism is associated with major depression but not with bipolar disorder. J Affect Disord.

[27] Fabbri C, Corponi F, Albani D, Raimondi I, Forloni G, Schruers K, Kasper S, Kautzky A, Zohar J, Souery D (2018). Pleiotropic genes in psychiatry: calcium channels and the stress-related FKBP5 gene in antidepressant resistance. Prog Neuro-psychopharmacol Biol Psychiatry.

[28] Zobel A, Schuhmacher A, Jessen F, Hfels S, Von Widdern O, Metten M, Pfeiffer U, Hanses C, Becker T, Rietschel M (2010). DNA sequence variants of the FKBP5 gene are associated with unipolar depression. Int J Neuropsychopharmacol.

[29] Cattaneo A, Gennarelli M, Uher R, Breen G, Farmer A, Aitchison KJ, Craig IW, Anacker C, Zunsztain PA, McGuffin P (2013). Candidate genes expression profile associated with antidepressants response in the GENDEP study: differentiating between baseline ‘predictors’ and longitudinal ‘targets’. Neuropsychopharmacology.

[30] Lekman M, Laje G, Charney D, Rush AJ, Wilson AF, Sorant AJ, Lipsky R, Wisniewski SR, Manji H, McMahon FJ (2008). The FKBP5-gene in depression and treatment response–an association study in the Sequenced Treatment Alternatives to Relieve Depression (STAR*D) cohort. Biol Psychiatry.

[31] Kirchheiner J, Lorch R, Lebedeva E, Seeringer A, Roots I, Sasse J, Brockmöller J (2008). Genetic variants in FKBP5 affecting response to antidepressant drug treatment. Pharmacogenomics.

[32] Binder EB, Salyakina D, Lichtner P, Wochnik GM, Ising M, Putz B, Papiol S, Seaman S, Lucae S, Kohli MA (2004). Polymorphisms in FKBP5 are associated with increased recurrence of depressive episodes and rapid response to antidepressant treatment. Nat Genet.

[33] Gawlik MMEK, Mende M (2006). Is FKBP5 a genetic marker of affective psychosis? A case control study and analysis of disease related traits. BMC Psychiatry.

[34] Yan Y, Jingping Z, Dong Y (2012). Non-association between tacrolimus binding protein 5 gene polymorphisms and major depressive disorder in Chinese Han population. Chin J Psychiatry.

[35] Papiol S, Arias B, Gasto C, Gutierrez B, Catalan R, Fananas L (2007). Genetic variability at HPA axis in major depression and clinical response to antidepressant treatment. J Affect Disord.

[36] Tsai SJ, Hong CJ, Chen TJ, Yu YW (2007). Lack of supporting evidence for a genetic association of the FKBP5 polymorphism and response to antidepressant treatment. Am J Med Genet B Neuropsychiatr Genet.

[37] Yang C, Li S, Ma Y, Chen B, Li M, Bosker FJ, Li J, Nolte IM. Lack of association of FKBP5 SNPs and haplotypes with susceptibility and treatment response phenotypes in Han Chinese with major depressive disorder A pilot case-control study (STROBE). Medicine 2021, 100(36). 10.1097/MD.0000000000026983 PMC8428740 34516490

[38] Ising M, Maccarrone G, Brückl T, Scheuer S, Hennings J, Holsboer F, Turck CW, Uhr M, Lucae S (2019). FKBP5 gene expression predicts antidepressant treatment outcome in Depression. Int J Mol Sci.

[39] Uher R, Huezo-Diaz P, Perroud N, Smith R, Rietschel M, Mors O, Hauser J, Maier W, Kozel D, Henigsberg N (2009). Genetic predictors of response to antidepressants in the GENDEP project. Pharmacogenom J.

[40] Stamm TJ, Rampp C, Wiethoff K, Stingl J, Moessner R, O’Malley G, Ricken R, Seemueller F, Keck M, Fisher R (2016). The FKBP5 polymorphism rs1360780 influences the effect of an algorithm-based antidepressant treatment and is associated with remission in patients with major depression. J Psychopharmacol.

[41] Dong Y, Jingping Z, Yan Y, Renrong W, Wenbin G (2013). Tacrolimus binding proteins 5 gene polymorphisms in major depressive disorder. Chin J Psychiatry.

[42] Sarginson JE, Lazzeroni LC, Ryan HS, Schatzberg AF, Murphy GM (2010). FKBP5 polymorphisms and antidepressant response in geriatric depression. Am J Med Genet B Neuropsychiatr Genet.

[43] Ellsworth KA, Moon I, Eckloff BW, Fridley BL, Jenkins GD, Batzler A, Biernacka JM, Abo R, Brisbin A, Ji Y (2013). FKBP5 genetic variation: association with selective serotonin reuptake inhibitor treatment outcomes in major depressive disorder. Pharmacogenet Genomics.

[44] Brent D, Melhem N, Ferrell R, Emslie G, Wagner KD, Ryan N, Vitiello B, Birmaher B, Mayes T, Zelazny J (2010). Association of FKBP5 polymorphisms with suicidal events in the treatment of resistant depression in adolescents (TORDIA) study. Am J Psychiatry.

[45] Perroud N, Bondolfi G, Uher R, Gex-Fabry M, Aubry J-M, Bertschy G, Malafosse A, Kosel M (2011). Clinical and genetic correlates of suicidal ideation during antidepressant treatment in a depressed outpatient sample. Pharmacogenomics.

[46] Nobile B, Ramoz N, Jaussent I, Dubois J, Guillaume S, Gorwood P, Courtet P (2020). Polymorphisms of stress pathway genes and emergence of suicidal ideation at antidepressant treatment onset. Transl Psychiatry.

[47] Ioannidis JP, A NEE, Trikalinos TA (2001). Replication validity of genetic association studies. Nat Genet.

[48] Natale A, Mineo L, Fusar-Poli L, Aguglia A, Rodolico A, Tusconi M, Amerio A, Serafini G, Amore M, Aguglia E (2022). Mixed depression: a mini-review to guide clinical practice and future research developments. Brain Sci.

[49] Fabbri C, Crisafulli C, Calabrò M, Spina E, Serretti A (2016). Progress and prospects in pharmacogenetics of antidepressant drugs. Expert Opin Drug Metab Toxicol.

[50] Singh AB, Bousman CA (2017). Antidepressant pharmacogenetics. Am J Psychiatry.

[51] Lett TA, Walter H, Brandl EJ (2016). Pharmacogenetics and imaging-pharmacogenetics of antidepressant response: towards translational strategies. CNS Drugs.

[52] Tozzi L, Farrell C, Booij L, Doolin K, Nemoda Z, Szyf M, Pomares FB, Chiarella J, O’Keane V, Frodl T (2018). Epigenetic changes of FKBP5 as a link connecting genetic and environmental risk factors with structural and functional brain changes in Major Depression. Neuropsychopharmacology.

[53] Budzinski ML, Sokn C, Gobbini R, Ugo B, Antunica-Noguerol M, Senin S, Bajaj T, Gassen NC, Rein T, Schmidt MV (2022). Tricyclic antidepressants target FKBP51 SUMOylation to restore glucocorticoid receptor activity. Mol Psychiatry.

[54] Busch Y, Menke A (2019). Blood-based biomarkers predicting response to antidepressants. J Neural Transm.

[55] Kwon A, Kim S, Jeon H, Lee HS, Lee SH (2021). Influence of FKBP5 variants and Childhood Trauma on Brain volume in non-clinical individuals. Front Behav Neurosci.

[56] Gałecki P, Samochowiec J, Mikułowska M, Szulc A (2022). Treatment-resistant depression in Poland—Epidemiology and Treatment. J Clin Med.

